# Clinical Trial Adaptation by Matching Evidence in Complementary Patient Sub-groups of Auxiliary Blinding Questionnaire Responses

**DOI:** 10.1371/journal.pone.0131524

**Published:** 2015-07-10

**Authors:** Ognjen Arandjelović

**Affiliations:** Centre for Pattern Recognition and Data Analytics, School of Information Technology, Deakin University, Geelong, Victoria, Australia; Baylor College of Medicine, UNITED STATES

## Abstract

Clinical trial adaptation refers to any adjustment of the trial protocol after the onset of the trial. Such adjustment may take on various forms, including the change in the dose of administered medicines, the frequency of administering an intervention, the number of trial participants, or the duration of the trial, to name just some possibilities. The main goal is to make the process of introducing new medical interventions to patients more efficient, either by reducing the cost or the time associated with evaluating their safety and efficacy. The principal challenge, which is an outstanding research problem, is to be found in the question of how adaptation should be performed so as to minimize the chance of distorting the outcome of the trial. In this paper we propose a novel method for achieving this. Unlike most of the previously published work, our approach focuses on trial adaptation by sample size adjustment i.e. by reducing the number of trial participants in a statistically informed manner. We adopt a stratification framework recently proposed for the analysis of trial outcomes in the presence of imperfect blinding and based on the administration of a generic auxiliary questionnaire that allows the participants to express their belief concerning the assigned intervention (treatment or control). We show that this data, together with the primary measured variables, can be used to make the probabilistically optimal choice of the particular sub-group a participant should be removed from if trial size reduction is desired. Extensive experiments on a series of simulated trials are used to illustrate the effectiveness of our method.

## Introduction

Robust evaluation is a crucial component in the process of introducing new medical interventions. Amongst others, these include newly developed medications, novel means of administering known treatments, new screening procedures, diagnostic methodologies, physio-therapeutical manipulations, and many others. Such evaluations usually take on the form of a controlled clinical trial (or a series thereof), the framework widely accepted as best suited for a rigourous statistical analysis of the effects of interest [1–3] (for a related discussion and critique also see [4]). Driven both by legislating bodies, as well as the scientific community and the public, the standards that the assessment of novel interventions are expected to meet continue to rise. Generally, this necessitates trials which employ larger sample sizes and which perform assessment over longer periods of time. A series of practical challenges emerge as a consequence. Increasing the number of individuals in a trial can be difficult because some trials necessitate that participants meet specific criteria; volunteers are also less likely to commit to participation over extended periods of time. The financial impact is another major issue—both the increase in the duration of a trial and the number of participants result in additional cost to an already expensive process. In response to these challenges, the use of adaptive trials has emerged as a potential solution [5–9].

The key idea underlying the concept of an adaptive trial design is that instead of fixing the parameters of a trial before its onset, greater efficiency can be achieved by adjusting them as the trial progresses [10]. For example, the trial sample size (e.g. the number of participants in a trial), treatment dose or frequency, or the duration of the trial may be increased or decreased depending on the accumulated evidence [11–13].

### Proposed method overview

The method for trial adaptation we describe in this paper extends the analysis presented in [14] which has been greatly influenced by recent work on the analysis of imperfectly blinded clinical trials [15, 16]. Its key contribution was to introduce the idea of trial outcome analysis by patient sub-groups which comprise trial participants matched by the administered intervention (treatment or control) and their responses to an auxiliary questionnaire in which the participants are asked to express their belief regarding their assignment intervention in the closed-form (see *Auxiliary data collection* for a summary of the adopted sub-group stratification method and the original paper [16] for full detail). This framework was shown to be suitable for robust inference in the presence of “unblinding” in a trial [16, 17]. The method proposed in the present paper emerges from the realization that the same framework can be used for trial adaptation by providing information which can be used to make a statistically informed selection of the trial participants which can be dropped from the trial before its completion, without significantly affecting the trial outcome. Thus, the proposed approach falls under the category of trial adaptations by “amending sample size”, in contrast to “dose finding” or “response adapting” methods which dominate previous work [13].

In [16] it was shown that the analysis of a trial’s outcome should be performed by aggregating evidence provided by matched participant sub-groups, where two sub-groups are matched if they contain participants who were administered different interventions but nonetheless had the same responses in the auxiliary questionnaire. Therefore, our idea advanced here is that an informed trial sample size reduction can be made by computing which matched sub-group pair’s contribution of useful information is affected the least with the removal of a certain number of participants from one of its groups.

#### Contrast with previous work

Before introducing the proposed method in detail, it is worthwhile emphasizing two fundamental aspects in which it differs from the methods previously described in the literature. The first difference concerns the nature of the statistical framework which underlies our approach. While the use of Bayesian techniques has become increasingly common in medical statistics [18–22], and perhaps particularly so in the context of clinical trial design and analysis [18, 23–28], with few notable exceptions [29] the existing work on trial adaptation by sample size adjustment adopts the frequentist paradigm [30–32]. These methods follow the following general pattern: a particular null hypothesis is formulated which is then rejected or accepted using a suitable statistic and the desired confidence requirement (a good review is provided by Jennison and Turnbull [33]). In contrast, the method described in this paper is thoroughly Bayesian in nature.

The second major conceptual novelty of the proposed method lies in the question it seeks to answer. Previous work on trial adaptation by sample size adjustment addresses the question of *whether* the sample size can be reduced while maintaining a certain level of statistical significance of the trial’s outcome. In contrast, the present work is the first to ask a complementary question of *which* particular individuals in the sample should be removed from the trial once the decision of sample size reduction has been made. Thus, the proposed method should not be seen as an alternative to the any of the previously proposed methods but rather as a complementary element of the same framework.

### Auxiliary data collection

The type of auxiliary data collection we utilize in this work was originally proposed for the assessment of blinding in clinical trials [34]. Since then it has been adopted for the same purpose in a number of subsequent works [16, 35–37] (also see [38] for related commentary).

With the exception of [16], in all previous work the questionnaire is employed after the trial has ended (for discussions on the timing of the questionnaire see [16, 39, 40]). The questionnaire allows the trial participants to express their belief on the nature of the intervention they have been administered (control or treatment) using a fixed number of choices. The most commonly used, coarse-grained questionnaire admits the following three choices:

Choice 1: the patient believes that he/she was administered the control intervention (i.e. control group membership),
Choice 2: the patient believes that he/she was administered the treatment intervention (i.e. treatment group membership), and
Choice 3: the patient is undecided about the nature of the treatment he/she was administered (the “don’t know” response).

Extensions of this scheme which attempt to harness more detailed information have also been used, for example allowing the participants to quantify the conviction of their belief as “weak” or “strong”. In that case, the questionnaire would offer five choices:

Choice 1: the patient *strongly* believes that he/she was administered the control intervention,
Choice 2: the patient *weakly* believes that he/she was administered the control intervention,
Choice 3: the patient is undecided about the nature of the treatment he/she was administered,
Choice 4: the patient *weakly* believes that he/she was administered the treatment intervention, and
Choice 5: the patient *strongly* believes that he/she was administered the treatment intervention.

More granular auxiliary data choices have the potential of providing a more accurate picture of the extent of blinding. However, depending on the statistical model used, this advantage may come at the cost of reduced statistical significance for each of the response sub-groups. The evidence on the capacity of a human’s working memory [41] suggests that the number of comparisons a typical person can distinguish at any one time is approximately 7±2, which leads to the conclusion that it is best to limit the number of choices to fewer than ten.

### Matching sub-groups outcome model

In the general case, the effectiveness of a particular intervention in a trial participant depends on the inherent effects of the intervention, as well as the participant’s expectations (conscious or not). Thus, as in [16], in the interpretation of trial results, we separately consider each population of participants which share the same combination of the type of intervention and the expressed belief regarding this group assignment. For example, when a 3-tier questionnaire is used in a trial comparing the administration of the treatment of interest and control, we recognize 3×2 = 6 auxiliary data sub-groups:

Sub-group 1: participants of the control group who believe they were assigned to the control group (sub-group *G*_*C*−_),
Sub-group 2: participants of the control group who are unsure of their group assignment (sub-group *G*_*C*0_),
Sub-group 3: participants of the control group who believe they were assigned to the treatment group (sub-group *G*_*C*+_),
Sub-group 4: participants of the treatment group who believe they were assigned to the control group (sub-group *G*_*T*−_),
Sub-group 5: participants of the treatment group who are unsure of their group assignment (sub-group *G*_*T*0_), and
Sub-group 6: participants of the treatment group who believe they were assigned to the treatment group (sub-group *G*_*T*+_).

**Fig 1:**
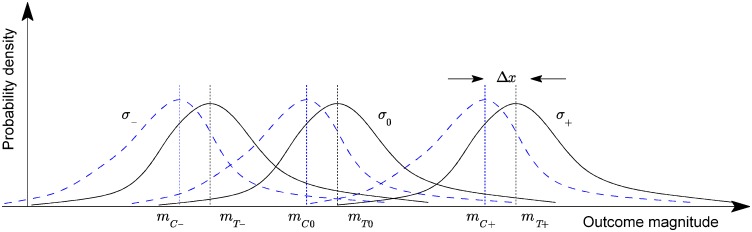
Adopted statistical model for a three-tier feedback questionnaire (conceptual illustration)—the probability densities of the measured trial outcome across the three control (solid lines) and treatment sub-groups (dotted lines). Diagram reproduced with permission.

This is illustrated conceptually in Fig 1. In the general case, for an *N*-tier questionnaire and *M* different intervention types, we can distinguish between *N*×*M* distinct sub-groups of participants.

The key idea underlying the method proposed in [16] is that because the outcome of an intervention depends on both the inherent effects of the intervention and the participants’ expectations, the effectiveness should be inferred in a like-for-like fashion. In other words, the response observed in, say, the sub-group of participants assigned to the control group whose feedback professes belief in the control group assignment should be compared with the response of only the sub-group of the treatment group who equally professed belief in the control group assignment. Similarly, the “don’t know” sub-groups should be compared only with each other, as should the sub-groups corresponding to the belief in the treatment assignment. Ideas similar in spirit were expressed by Berger in the consideration of the related problem of so-called selection bias and specifically the Berger-Exner test [42].

## Sub-group selection

The primary aim of the statistical framework described in [16] is to facilitate an analysis of trial data robust to the presence of partial or full unblinding of patients, or indeed patient preconceptions which too may affect the measured outcomes. Herein we propose to exploit and extend this framework to guide the choice of which patients are removed from the trial after its onset, in a manner which minimizes the loss of statistical significance of the ultimate outcomes.

At the onset of the trial, the trial should be randomized according to the current best clinical practice; this problem is comprehensively covered in the influential work by Berger [42]. If a reduction in the number of trial participants was attempted at this stage, by the very definition of a properly randomized trial, statistically speaking there is no reason to prefer the removal of any particular subject (or indeed a set of subjects) over another. Instead, any trial size adaptation must be performed at a later stage after some meaningful differentiation between subjects takes place [43–45].

The most obvious measurable differentiation that takes place between patients as the trial progresses is that of the outcomes of primary interest in the trial (the “response”). This differentiation may allow for a statistically informed choice to be made about which trial participants can be dropped from the trial in a manner which minimizes the expected distortion of the ultimate findings. For example, this can be done by seeking to preserve the distribution of measured outcomes within a group (treatment or control) but with the constraint of a smaller number of participants; indeed, our approach partially exploits this idea as will be explained in detail shortly. However, our key contribution lies in a more innovative approach, which exploits additional, yet readily collected discriminative information. The proposed approach not only minimizes the effect of smaller participant groups but also ensures that no unintentional bias is injected due to imperfect blinding. Recall that the problem of inference robust to imperfect blinding should always be considered—as stated earlier, blinding can only be attempted with respect to those variables of the trial which have been identified as revealing of the administered treatment, and even for the explicitly identified variables it is fundamentally impossible to ensure that absolute blinding is achieved.

**Fig 2:**
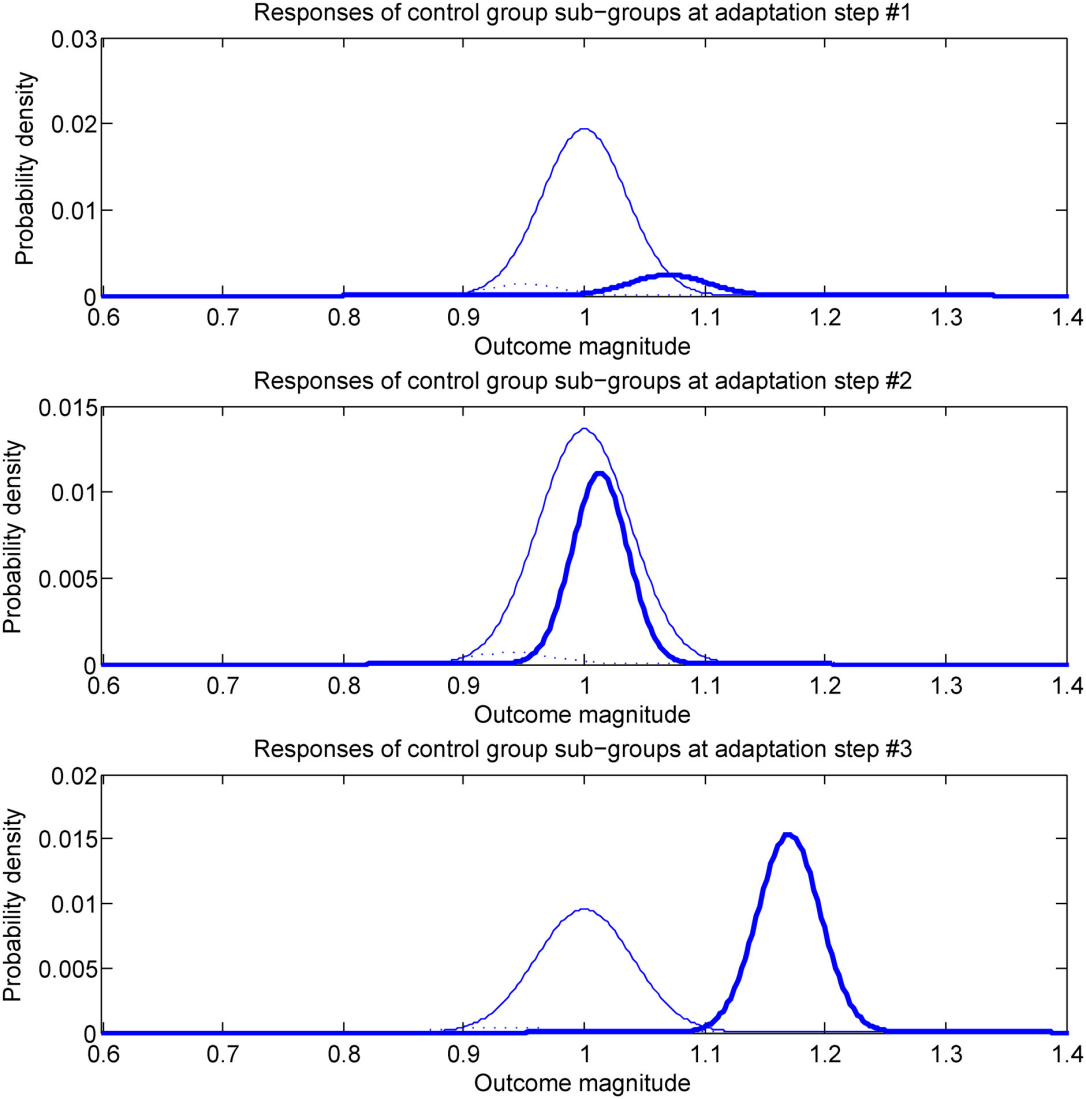
A conceptual illustration on a hypothetical example of the phenomenon whereby trial participants change their sub-group membership (recall that each sub-group is defined by its members’ intervention assignment *and* auxiliary questionnaire responses). This is quite likely to occur when the effects of the treatment are very readily apparent but various other mechanisms can act so as to cause a non-zero and changing sub-group flux.

Our idea is to administer an auxiliary questionnaire of the form described in [34, 35] (which is normally administered after the trial in the work on blinding assessment) every time an adaptation of the trial group size (i.e. reduction thereof) is sought. Just like in [16], this leads to the differentiation of each group of participants (control or treatment) into sub-groups, based on their belief regarding their group assignment. In general, this means that even if no participants are removed from the trial, a participant may change his/her sub-group membership status. This is illustrated conceptually in Fig 2. The first time an auxiliary questionnaire is administered (top-most plot), most of the treatment group participants are still unsure of their assignment (solid blue line); a smaller number of participants have correctly guessed (or inferred) their assignment (bold blue line); lastly, an even smaller number holds the incorrect belief that they are in fact members of the control group (dotted blue line). All of the sub-groups show a spread of responses to the treatment, such as may be expected due to various personal variations of their members. At the time of the second snapshot (plot in the middle), at the next instance when auxiliary data is collected, the proportions of participants in each sub-group has changed, as do the associated treatment response statistics. A similar observation can be made with respect to the third and the last snapshot pictured in the figure (bottom-most plot). This sort of a development would not be unexpected—if the treatment is effective, as the trial progresses there will be an increase in the number of treatment group participants who observe and correctly interpret these changes (note that this also means that there will be an associated increase in the number of participants who may exhibit an additional positive effect from the fortunate realization that they are receiving the studied treatment intervention, rather than the control intervention). That being said, it should be emphasized that no assumption on the statistics of sub-group memberships or their relative sizes is made in the proposed method. The example in Fig 2 is merely used for illustrative purposes.

The question is: how does this differentiation of patients by auxiliary data sub-groups help us make a statistically robust choice of which participants in the trial should be preferentially dropped if a reduction in the trial size is sought? To answer this question, recall the main premise of [16]:

“The key idea […] is that it is meaningful to compare only the corresponding treatment and control participant sub-groups, that is, sub-groups matched by their auxiliary responses.”

Each sub-group comparison contributes information used to infer the probability density of the differential effects of the treatment. We can then reformulate the original question as: from which matching sub-group pair should participants be preferentially dismissed from further consideration so as to best preserve the information contribution from all sub-groups?

Consider how the information on the differential effects between a single pair of matching sub-groups is inferred. In its general form, we can estimate some distance between the distributions of the two sub-groups using a Bayesian approach. Indeed, Bayesian analysis based methods have in recent years been continually gaining acceptance across the clinical community [44]. In brief, the key idea is to “integrate out” the unknown latent parameters of the two distributions. Expressed formally:

$$\rho^{*}=\int_{\Theta_{c}}\int_{\Theta_{t}}\underset{\begin{array}{c} \text{Distance}\;\text{between}\;\text{distributions}\\ \text{for}\;\text{specific}\;\text{parameter}\;\text{values} \end{array}}{\underset{︸}{\rho (p_{c}\left(x;\Theta_{c}\right),p_{t}\left(x;\Theta_{t}\right))}}\times \underset{\begin{array}{c} \text{Probability}\;\text{of}\;\text{parameters}\\ \text{conditioned}\;\text{on}\;\text{observations} \end{array}}{\underset{︸}{p\left(\Theta_{c}|D_{c}\right)\;p\left(\Theta_{t}|D_{t}\right)}}\times \underset{\text{Parameter}\;\text{priors}}{\underset{︸}{p\left(\Theta_{c}\right)\;p\left(\Theta_{t}\right)}}\times \;d\Theta_{t}\;d\Theta_{c}$$ (1)

where Θ_*c*_ and Θ_*t*_ are the sets of variables parameterizing the two corresponding distributions *p*_*c*_(*x*;Θ_*c*_) and *p*_*t*_(*x*;Θ_*t*_), *p*(Θ_*c*_) and *p*(Θ_*t*_) the parameter priors, *ρ*(*p*_*c*_(*x*;Θ_*c*_),*p*_*t*_(*x*;Θ_*t*_)) a particular distance function (e.g. the Kullback-Leibler divergence [46], Bhattacharyya [47] or Hellinger distances [48], or indeed the posterior of the difference of means used in [16]), and *D*_*c*_ and *D*_*t*_ the measured trial outcomes (such as the amount of fat loss in a fat loss trial, the reduction in blood plasma LDL in a statin trial etc).

Note that by changing (reducing) the number of participants in one of the groups, the only affected term on the right hand side of Eq (1) is one of the likelihood terms, *p*(Θ_*c*_∣*D*_*c*_) or *p*(Θ_*t*_∣*D*_*t*_). Seen another way, a change in the number of participants in the trial changes the *weighting* of the product of the distance term *ρ*(*p*_*c*_(*x*;Θ_*c*_),*p*_*t*_(*x*;Θ_*t*_)) and the priors *p*(Θ_*c*_) *p*(Θ_*t*_). Our idea is then to choose to remove a trial participant from that sub-group which produces the smallest change in the estimate ρ*. However, it is not clear how this may be achieved, since it is the size of the set *D*_*c*_ that is changing (so, for example, treating *D*_*c*_ and *D*_*t*_ as vectors and *f* as a function of vectors would not achieve the desired aim). Examining the sensitivity of ρ* with the removal of each datum (i.e. trial participant) from *D*_*c*_ and *D*_*t*_ is also unsatisfactory since the problem does not lend itself to a greedy strategy: the optimal choice of which *n*_*rem*_ trial participants to drop from the trial cannot be made by making *n*_*rem*_ optimal choices of which *one* participant to drop. An approach following this direction but attempting to examine all possible sets of size *n*_*rem*_ would encounter computational tractability obstacles since this problem is NP-complete. The alternative which we propose is to consider and compare the magnitudes of partial derivatives of ρ* with respect to the sizes of data sets *D*_*c*_ and *D*_*t*_, but with an important constraint—the derivatives are taken of the *expected* functional form of ρ* over different members of *D*_*c*_ and *D*_*t*_. Formalizing this, we compute:

$$E{\left[\frac{\partial \rho^{*}}{\partial n_{c}}\right]}_{D_{c}}\;\text{and}\;E{\left[\frac{\partial \rho^{*}}{\partial n_{t}}\right]}_{D_{t}},$$ (2)

where *E*[ρ*]_*D*_*c*__ and *E*[ρ*]_*D*_*t*__ are respectively the expected values of ρ* across the space of possible observations in *D*_*c*_ and *D*_*t*_ (each with a uniform prior, as before). Thus *E*[ρ*]_*D*_*c*__ and *E*[ρ*]_*D*_*t*__ are functions of two scalars, the sizes *n*_*c*_ and *n*_*t*_ of sets *D*_*c*_ and *D*_*t*_ i.e. the numbers of members of the corresponding sub-groups.

The proposed solution is not only theoretically justified but it also lends itself to simple and efficient implementation. Since the expected values *E*[ρ*]_*D*_*c*__ and *E*[ρ*]_*D*_*t*__ are evaluated over sets *D*_*c*_ and *D*_*t*_, in Eq (1) the only term affected is *p*(Θ_*c*_∣*D*_*c*_) *p*(Θ_*t*_∣*D*_*t*_), so the solution is readily obtained as a closed form expression. Equally, the integration is readily performed using one of the standard Markov chain Monte Carlo integration methods [49].

## Application example

In order to illustrate how the described idea could be applied in practice, we now consider one specific example of the distance function discussed in the previous section and show how the mathematical results needed to implement the proposed methodology can be derived or estimated. Readers who are not interested in full technical detail of this nature can skip this section and proceed to *Note on practical application* without loss in continuity.

Let the trial observation data in two matching sub-groups be drawn from the random variables *X*_*c*_ and *X*_*t*_, which are appropriately modelled using log-normal distributions [50]:

$$X_{t}∼\frac{1}{x\;\sigma_{t}}\exp\{-\frac{\ln x-m_{t}}{2\sigma_{t}^{2}}\}$$ (3)

and

$$X_{c}∼\frac{1}{x\;\sigma_{c}}\exp\{-\frac{\ln x-m_{c}}{2\sigma_{c}^{2}}\}$$ (4)

The next step is to choose an appropriate distance function *ρ* in Eq (1). In practice, this choice would be governed by the goals of the study. Herein, for illustrative purposes we choose *ρ* to be the probability that a patient will do better when the treatment rather than the control intervention is administered:

$$\rho \left(p_{t}\left(x;\Theta_{t}\right),p_{c}\left(y;\Theta_{c}\right)\right)=\int_{0}^{\infty }\int_{0}^{x}p_{t}\left(x;\Theta_{t}\right)\;p_{c}\left(y;\Theta_{c}\right)\;dy\;dx$$

where Θ_*c*_ = (*m*_*c*_,*σ*_*c*_) and Θ_*t*_ = (*m*_*t*_,*σ*_*t*_) are the mean and standard deviation parameters specifying the corresponding log-normal distributions.

$$\begin{array}{l} \rho^{*}\propto \\ \;\int_{0}^{\infty }\int_{-\infty }^{\infty }\int_{0}^{\infty }\int_{-\infty }^{\infty }\int_{0}^{\infty }\int_{0}^{x}\left\{p_{t}(x\left|m_{t},\sigma_{t})\;p\left(m_{t},\sigma_{t}\right)\;dx\;dm_{t}\;d\sigma_{t}\;\times p_{c}(y\right|m_{c},\sigma_{c})\;p\left(m_{c},\sigma_{c}\right)\;dy\;dm_{c}\;d\sigma_{c}\right\} \end{array}$$ (5)

Making the usual substitution whereby *e* ^*x*^ is substituted for *x* (and similarly *e* ^*y*^ for *y*), which normalizes the log-normal distribution, and assuming uninformed priors on *m*_*c*_, *m*_*t*_, *σ*_*c*_, and *σ*_*t*_ (just as in [16]) leads to the following expression

$$\rho^{*}\propto \overset{\infty }{\underset{0}{\int }}\overset{\infty }{\underset{-\infty }{\int }}\overset{\infty }{\underset{0}{\int }}\overset{\infty }{\underset{-\infty }{\int }}\overset{\infty }{\underset{0}{\int }}\overset{x}{\underset{0}{\int }}\sigma_{t}^{-1}\;e^{-\frac{{\left(x-m_{t}\right)}^{2}}{2\sigma_{t}^{2}}}\;\sigma_{t}^{-N}\;e^{-\frac{\sum_{i=1}^{n_{t}}{\left(x_{i}^{\left(t\right)}-m_{t}\right)}^{2}}{2\sigma_{t}^{2}}}\;dx\;dm_{t}\;d\sigma_{t}\times \sigma_{c}^{-1}\;e^{-\frac{{\left(x-m_{c}\right)}^{2}}{2\sigma_{c}^{2}}}\;\sigma_{c}^{-N}\;e^{-\frac{\sum_{i=1}^{n_{c}}{\left(x_{i}^{\left(c\right)}-m_{c}\right)}^{2}}{2\sigma_{c}^{2}}}\;dy\;dm_{c}\;d\sigma_{c}$$ (6)

$$=\overset{\infty }{\underset{0}{\int }}\overset{x}{\underset{0}{\int }}I_{t}\left(x\right)\;I_{c}\left(y\right)\;dy\;dx=\overset{\infty }{\underset{0}{\int }}I_{t}\left(x\right)\overset{x}{\underset{0}{\int }}I_{c}\left(y\right)\;dy\;dx$$ (7)

where each of the integrals *I*_*t*_(*x*) and *I*_*c*_(*y*) has the form:

$$I=\int_{0}^{\infty }\int_{-\infty }^{\infty }\frac{1}{\sigma }\;\exp\{-\frac{{\left(x-m\right)}^{2}}{2\sigma^{2}}\}\;\times \frac{1}{\sigma^{n}}\;\exp\{-\frac{\sum_{i=1}^{n}{\left(x_{i}-m\right)}^{2}}{2\sigma^{2}}\}\;dm\;d\sigma ,$$ (8)

{*x*_*i*_} and *n* stand for either $\left\{x_{i}^{\left(c\right)}\right\}$ and *n*_*c*_ or $\left\{x_{i}^{\left(t\right)}\right\}$ and *n*_*t*_, and $\left\{x_{i}^{\left(c\right)}\right\}$ (*i* = 1…*n*_*c*_) and $\left\{x_{i}^{\left(t\right)}\right\}$ (*i* = 1…*n*_*t*_) are exponentially transformed measured trial variables. This integral can be evaluated by combining the two exponential terms and completing the square of the numerator of the exponent as in [16] so that:

$${\left(x-m\right)}^{2}+\sum_{i=1}^{n}{\left(x_{i}-m\right)}^{2}\equiv {\left(am+b\right)}^{2}+c,$$ (9)

which leads to the following simplification of Eq (7):

$$I\propto \overset{\infty }{\underset{0}{\int }}\frac{1}{\sigma^{n+2}}\;\text{exp}\left\{-\frac{c}{2\sigma^{2}}\right\}\;\sigma \;d\sigma$$ (10)

$$=\overset{\infty }{\underset{0}{\int }}\frac{1}{\sigma^{n+1}}\;\text{exp}\left\{-\frac{c}{2\sigma^{2}}\right\}\;d\sigma ,$$ (11)

where the value of the only non-constant term, *c*, is:

$$c=x^{2}+\overset{n}{\underset{i=1}{\sum }}x_{i}{}^{2}-\frac{{\left(x+\sum_{i=1}^{n}x_{i}\right)}^{2}}{n+1}$$ (12)

$$=\frac{\left(n+1\right)\left(x^{2}+\sum_{i=1}^{n}x_{i}{}^{2}\right)-{\left(x+\sum_{i=1}^{n}x_{i}\right)}^{2}}{n+1}.$$ (13)

Observing that the form of the integrand in Eq (11) matches that of the inverse gamma distribution:

$$\text{Gamma}\left(z;\alpha ,\beta \right)=\frac{\beta^{\alpha }}{\Gamma \left(\alpha \right)}\;z^{-\alpha -1}\;\text{exp}\left\{-\beta /z\right\}.$$ (14)

where Γ(*α*) is the value of the gamma function at *α*. The variable *z* and the two parameters of the distribution, *α* and *β*, can be matched with the terms in Eq (11) and the density integrated out, leaving the integral proportional to a single non-constant term:

$$I\propto c^{-\frac{n-1}{2}}.$$ (15)

Notice that the form of this result is very similar to that obtained in [16].

Remembering that the functional form of *c* is different for the control and the trial groups (since it is dependent on *x*_*i*_ which stands for either $x_{i}^{\left(c\right)}$ or $x_{i}^{\left(t\right)}$), and substituting the result from Eq (15) back into Eq (7) gives the following expression for the distance function:

$$\rho^{*}=\overset{\infty }{\underset{0}{\int }}\overset{x}{\underset{0}{\int }}p_{t}\left(x\right)\;p_{c}\left(y\right)\;dy\;dx$$ (16)

$$\propto \overset{\infty }{\underset{0}{\int }}\overset{x}{\underset{0}{\int }}c_{t}^{-\frac{n_{t}-1}{2}}c_{c}^{-\frac{n_{c}-1}{2}}\;dy\;dx$$ (17)

$$=\overset{\infty }{\underset{0}{\int }}c_{t}^{-\frac{n_{t}-1}{2}}\overset{x}{\underset{0}{\int }}c_{c}^{-\frac{n_{c}-1}{2}}\;dy\;dx$$ (18)

Our goal now is to evaluate *S*_*c*_(ρ*) and *S*_*t*_(ρ*), the sensitivities of the distance function to the change in the size of respectively the control and the treatment groups. Without loss of generality, let us consider *S*_*t*_(ρ*)—the symmetry of the expression in Eq (18) makes it trivial to apply the same process to the computation of *S*_*t*_(ρ*). From Eq (18):

$$S_{t}\left(\rho^{*}\right)\propto \overset{\infty }{\underset{-\infty }{\int }}\overset{x}{\underset{-\infty }{\int }}S\left[I_{t}\left(x\right)\right]\;I_{c}\left(y\right)\;dy\;dx$$ (19)

To evaluate *S*[*I*_*t*_(*x*)] we will employ the standard chain rule and perform differentiation with respect to *n*_*t*_ when the corresponding term is a function of the number of treatment participants but not any $x_{i}^{\left(t\right)}$. On the other hand, as proposed in *Sub-group selection*, to handle those terms which do depend on $x_{i}^{\left(t\right)}$ (through *c*_*t*_), we will use the expected value of the change in the term, averaged over all possible $x_{i}^{\left(t\right)}$ that a unitary decrease in *n*_*t*_ can be achieved. Applying this idea on the expression in Eq (8) leads to the following expansion:

$$S\left[I_{t}\left(x\right)\right]=-\overset{\infty }{\underset{0}{\int }}\overset{\infty }{\underset{-\infty }{\int }}\frac{1}{\sigma }\text{exp}\left\{-\frac{{\left(x-m\right)}^{2}}{2\sigma^{2}}\right\}\times \left[\frac{\text{ln}\sigma }{\sigma^{n}}\text{exp}\left\{-\frac{\sum_{i=1}^{n}{\left(x_{i}-m\right)}^{2}}{2\sigma^{2}}\right\}+\frac{1}{\sigma^{n}}\;\text{exp}\left\{-\frac{\sum_{i=1}^{n}{\left(x_{i}-m\right)}^{2}}{2\sigma^{2}}\right\}\;\frac{\sum_{i=1}^{n}{\left(x_{i}-m\right)}^{2}}{2\sigma^{2}n^{2}}\right]\;dm\;d\sigma$$ (20)

noting that we used the standard result:

$$\frac{d}{dn}\frac{1}{\sigma^{n}}=-\frac{\text{ln}\sigma }{\sigma^{n}}$$ (21)

without including its derivation with intermediary steps shown explicitly. The resulting expression can be further rearranged, yielding the following, more concise expression:

$$S\left[I_{t}\left(x\right)\right]=-\overset{\infty }{\underset{0}{\int }}\overset{\infty }{\underset{-\infty }{\int }}\frac{\text{ln}\sigma }{\sigma^{n}}\text{exp}\left\{-\frac{{\left(x-m\right)}^{2}}{2\sigma^{2}}\right\}\times \text{exp}\left\{-\frac{\sum_{i=1}^{n}{\left(x_{i}-m\right)}^{2}}{2\sigma^{2}}\right\}\;dm\;d\sigma$$ (22)

$$-\overset{\infty }{\underset{0}{\int }}\overset{\infty }{\underset{-\infty }{\int }}\frac{1}{\sigma^{n+3}n^{2}}\;\text{exp}\left\{-\frac{{\left(x-m\right)}^{2}}{2\sigma^{2}}\right\}\times \text{exp}\left\{-\frac{\sum_{i=1}^{n}{\left(x_{i}-m\right)}^{2}}{2\sigma^{2}}\right\}\overset{n}{\underset{i=1}{\sum }}{\left(x_{i}-m\right)}^{2}\;dm\;d\sigma .$$ (23)

Full double integration of the integrands in Eq (23) is difficult to perform analytically. However, one level of integration—with respect to *m*—is readily achieved. Note that the first term, as a function of *m*, has the same form as the integral in Eq (8) which we already evaluated. The same procedure which uses the completion of the square in the exponential term can be applied here as well (note that unlike in Eq (8) here it is important to keep track of the multiplicative constants as these will be different for the second term in Eq (23)). The integrand in the second term can be expressed in the form ∝ (*z*−*λ*)^2^ exp−*z* ^2^ *dz*. This integration is also readily performed using the standard results:

$$\overset{\infty }{\underset{-\infty }{\int }}\frac{1}{\sqrt{2\pi }}\;z^{2}\;\text{exp}-\frac{z^{2}}{2}\;dz=1$$ (24)

and

$$\overset{\infty }{\underset{-\infty }{\int }}\frac{1}{\sqrt{2\pi }}\;\text{exp}-\frac{z^{2}}{2}\;dz=1,$$ (25)

and by noting that the integrand is an odd function

$$\overset{\infty }{\underset{-\infty }{\int }}\frac{1}{\sqrt{2\pi }}\;z\;\text{exp}-\frac{z^{2}}{2}\;dz=0$$ (26)

A straightforward application of these results to Eq (23) leads to the following expression for the sensitivity *S*[*I*_*t*_(*x*)] of the integral *I*_*t*_ to changes in the size of the corresponding sub-group (the treatment sub-group in a matching pair):

$$S\left[I_{t}\left(x\right)\right]=-\overset{\infty }{\underset{0}{\int }}\frac{\text{ln}\sigma }{\sigma^{n+1}}\;\frac{\sigma }{a}\;\sqrt{2\pi }\;\text{exp}\left\{-\frac{c}{2\sigma^{2}}\right\}\;d\sigma -\overset{\infty }{\underset{0}{\int }}\frac{\text{exp}\left\{-\frac{c}{2\sigma^{2}}\right\}}{2\sigma^{n+3}n^{2}}\;\left[n{\left(\frac{\sigma }{a}\right)}^{3}+\sqrt{2\pi }\frac{\sigma }{a}\overset{n}{\underset{i=1}{\sum }}x_{i}\right]\;d\sigma$$ (27)

This result, together with the expression in Eq (15), can be substituted into Eq (19) and the remaining integration performed numerically.

### Note on practical application

As demonstrated in the preceding section, it is worth observing that none of the calculations involved make any strong assumptions on the nature of the phenomenon studied i.e. the nature of the outcome data. There are only two elements of the described framework which are subject to change depending on the trial. These are the distance function *ρ* and the form of the likelihood terms *p*(Θ_*c*_∣*D*_*c*_) and *p*(Θ_*t*_∣*D*_*t*_), see Eq (1). In practice, all of these terms are confined to a small number of reasonable options, which means that all of the derivations described in some detail in the previous section can be performed *a priori*, with only the final numerical computations (which are not computationally demanding) performed “on the fly”. Consequently, while the implementation of the proposed approach clearly requires expertise in mathematics and computation, no mathematical expertise or thorough understanding of the details of the algorithm need to be expected from the user (e.g. a clinician or a biostatistician). The user would merely need to select one of a number of possible outcomes of interest (e.g. “effect greater than”) and set the value of the relevant parameter according to the aims of the study (e.g. “effect greater than 0.1”), and be automatically presented with the best sample selection and the corresponding posterior distributions quantifying the uncertainty associated with the resultant outcome. This will be illustrated shortly on simulated examples in *Evaluation and analysis*.

### From target sub-groups to specific participants

Adopting the framework proposed in [16] whereby the analysis of a trial takes into account sub-groups of trial participants, which emerge from grouping participants according to their assigned intervention and auxiliary data, thus far we focused on the problem of choosing the sub-group from which participants should be preferentially removed if a reduction in trial size is sought. The other question which needs to be considered is how *specific* sub-group members are to be chosen, once the target sub-group is identified. Fortunately, the proposed framework makes this a simple task. Recall that the observed trial data within each sub-group is assumed to comprise an identically and independently distributed sample from the underlying distribution, i.e. $x_{i}^{\left(c\right)}∼X_{c}$ (or indeed $x_{i}^{\left(t\right)}∼X_{t}$). This means that it is sufficient to randomly sample the set of target sub-group members to select those which can be removed.

**Fig 3:**
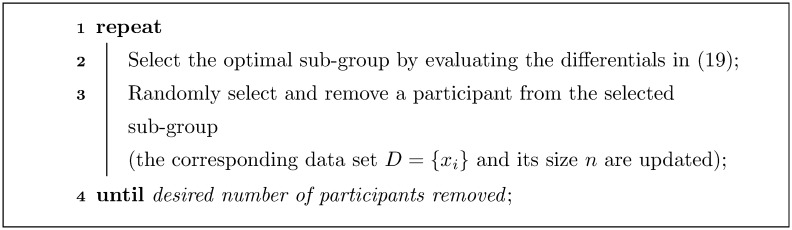
A ‘high-level’ summary of the key steps in the proposed method.

The simplicity of the selection process that our approach allows has an additional welcome consequence. Recall that in the proposed method the choice of the target sub-group is made by comparing differentials in Eq (2). It is important to observe that their values are computed for the initial values of *n*_*c*_ and *n*_*t*_. Thus, as the number of participants in either of the sub-groups is changed, so do the values of the differentials, and thus possibly the optimal sub-group choice. This is why the removal of participants should proceed sequentially as summarized in Fig 3.

## Evaluation and Analysis

The primary novelty introduced in this paper is of a methodological nature. In the previous section we explained in detail the mathematical process involved in applying the proposed methodology in practice. Pertinent results were derived for a specific distance function used to quantify the difference in the outcomes between the control and treatment groups in a trial. The choice of the distance function—which would in practice be made by the clinicians to suit the aims of a specific trial—governs the relative loss of information when participants are removed from a specific sub-group, and consequently dictates the choice of the optimal sub-group from which the removal should be performed if the overall trial sample size needs to be reduced.

In this section we apply the derived results on experimental data, and evaluate and discuss the performance of the proposed methodology. We adopt the evaluation protocol standard in the domain of adaptive trials research, and obtain data using a simulated experiment.

### Experimental setup

We simulated a trial involving 180 individuals, half of which were assigned to the control and the other half to the treatment group. For each individual we maintain a variable which describes that person’s belief regarding his/her group assignment. Thus, for the control group we have *n*_*c*_ beliefs $\left\{b_{i}^{\left(c\right)}\right\}$ (*i* = 1…*n*_*c*_) and similarly for the treatment group *n*_*t*_ beliefs $\left\{b_{i}^{\left(t\right)}\right\}$ (*i* = 1…*n*_*t*_). Belief is expressed by a real number, $\forall i.\;b_{i}^{\left(c\right)},b_{i}^{\left(t\right)}\in (-\infty ,+\infty )$, with 0 indicating true undecidedness. Negative beliefs express a preference towards the belief in control group assignment, and positive towards the belief in treatment group assignment. The greater the absolute value of a belief variable is, the greater is the person’s conviction. We employ a three-tier questionnaire. To simulate a participant’s response, we map the corresponding belief to one of the three possible questionnaire responses according to the following thresholding rule:

b<−1→Beliefincontrolgroupassignment (28)

−1≤b≤1→Uncertain(“don’tknow”) (29)

1<b→Beliefintreatmentgroupassignment (30)

The starting beliefs of participants, i.e. their beliefs before the onset of the trial, are initialized as follows:

$$b_{i}^{\left(c\right)}=b_{i}^{\left(t\right)}\{\begin{array}{ccc} -1 & \text{for} & i=1\ldots 8\;\;\;\;\;\;\\ 0 & \text{for} & i=9\ldots 81\;\;\;\\ 1 & \text{for} & i=82\ldots 90 \end{array}$$ (31)

Put in informal terms, this initialization reflects the conservative belief of most individuals, and the tendency of a smaller number of individuals to exhibit either “pessimistic” or “optimistic” expectations. Also, notice that the same distribution was used both for the control and the treatment groups, reflecting a well performed randomization in the group assignment process.

#### Effect accumulation

As the trial progresses the effects of the treatment accumulate. These are modelled as positive i.e. the treatment is modelled as successful in the sense that on average it produces a superior outcome in comparison with the control intervention. We model this using a stochastic process which captures the variability in participants’ responses to the same treatment. Specifically, at the discrete time step *k*+1 (the onset of the trial corresponding to *k* = 0), the effect of the *i*-th treatment group participant at the preceding time step *k*, $e_{i}^{\left(c\right)}$(k), is updated in the following manner:

$$e_{i}^{\left(t\right)}\left(k+1\right)=e_{i}^{\left(t\right)}\left(k\right)+w_{i}^{\left(t\right)}\left(k+1\right)\times \text{exp}\left\{-\frac{k+1}{10}\right\}$$ (32)

where $w_{i}^{\left(t\right)}(k+1)$ is drawn from a normal distribution:

$$W_{t}∼N\left(0.02,0.05\right).$$ (33)

Notice that this progression has a ‘ground truth’ asymptote at:

$$\underset{k\to \infty }{\text{lim}}E\left[e_{i}^{\left(t\right)}\left(k\right)\right]=e_{i}^{\left(t\right)}\left(0\right)+0.02\times \frac{\text{exp}\left\{-\frac{1}{10}\right\}}{1-\text{exp}\left\{-\frac{1}{10}\right\}}\approx e_{i}^{\left(t\right)}\left(0\right)+0.19$$ (34)

Similarly, for the control group participants:

$$e_{i}^{\left(c\right)}\left(k+1\right)=e_{i}^{\left(c\right)}\left(k\right)+w_{i}^{\left(c\right)}\left(k+1\right)\times \text{exp}\left\{-\frac{k+1}{10}\right\}$$ (35)

where $w_{i}^{\left(c\right)}(k+1)$ is drawn from a normal distribution:

$$W_{c}∼N\left(0.00,0.05\right)$$ (36)

By definition, at the onset of the trial there is no effect of the treatment; thus:

$$\forall .i=1\ldots n_{t}.\;e_{i}^{\left(t\right)}\left(0\right)=0$$ (37)

$$\forall .i=1\ldots n_{c}.\;e_{i}^{\left(c\right)}\left(0\right)=0$$ (38)

#### Belief refinement

As the effects of the respective interventions are exhibited, the trial participants have increasing amounts of evidence available guiding them towards forming the correct belief regarding their group assignment. In our experiment this process is also modelled using a stochastic process which is dependent on the magnitude of the effect that an intervention has in a particular participant, as well as uncertainty and differences in people’s inference from observations. At the discrete time step *k*+1, the belief of the *i*-th control group participant previous at the time step *k*, $b_{i}^{\left(c\right)}$(k), is updated using the following update equation:

$$b_{i}^{\left(c\right)}\left(k+1\right)=b_{i}^{\left(c\right)}\left(k\right)+\phi \times e_{i}^{\left(c\right)}\left(k+1\right)+\omega_{i}^{\left(c\right)}\left(k+1\right)$$ (39)

where $\omega_{i}^{\left(c\right)}(k+1)$ is drawn from a normal distribution:

Ω∼N(0.00,0.005). (40)

The model parameter *ϕ* captures the ease with which an improvement (or equivalently, a deterioration) in the outcome is observed by a patient. For example in a trial where the outcome of interest is, say, muscular strength or mobility [51], observability is high. In contrast, in a trial which examines the effects of different interventions on, say, bone mineral density [52], the patient is entirely or virtually entirely unable to gauge relevant changes. As expected from the theory presented in the previous sections and as we shall demonstrate empirically, this aspect of a clinical trial under consideration has important consequences on the benefit of sample selection proposed in this work. This is investigated in detail in the next section.

The changes in the beliefs of the treatment group are modelled in the identical manner to those of control group participants:

$$b_{i}^{\left(t\right)}\left(k+1\right)=b_{i}^{\left(t\right)}\left(k\right)+\phi \times e_{i}^{\left(t\right)}\left(k+1\right)+\omega_{i}^{\left(t\right)}\left(k+1\right)$$ (41)

where $\omega_{i}^{\left(t\right)}(k+1)$ is again drawn from a normal distribution:

$$\Omega_{c}∼N\left(0.00,0.005\right)$$ (42)

### Experiment 1: Baseline performance

Using the model for generating longitudinal patient data described in *Experimental setup*, in this experiment we compared the proposed method with random sample selection in the context of a single sample size reduction step. In particular we simulated the reduction in the total number of participants of *n* = 70 (i.e. approximately 39% of the total cohort size of 180) after *k* = 50 time steps; please see Eqs (32)–(41). Following the removal time step, data accrual of the selected participants was discontinued. Note that in this experiment the data collected from the removed patients up to the time step *k* = 50 *was* used in the final outcome analysis. The trial was simulated for the total number of *k*_max_ = 100 time steps after which the outcomes were used to infer the differential effects of the two simulated interventions. The final outcomes both in the case of the proposed method and random selection were analysed using the Bayesian method proposed in [16]. The observability parameter in this experiment was set to *ϕ* = 0.01, please see Eq (39). Finally, to facilitate robust analysis, the experiment was repeated 100 times, resulting in different patient states, effects of the two interventions (treatment and control), as well as different removal choices for both selection procedures.

**Fig 4:**
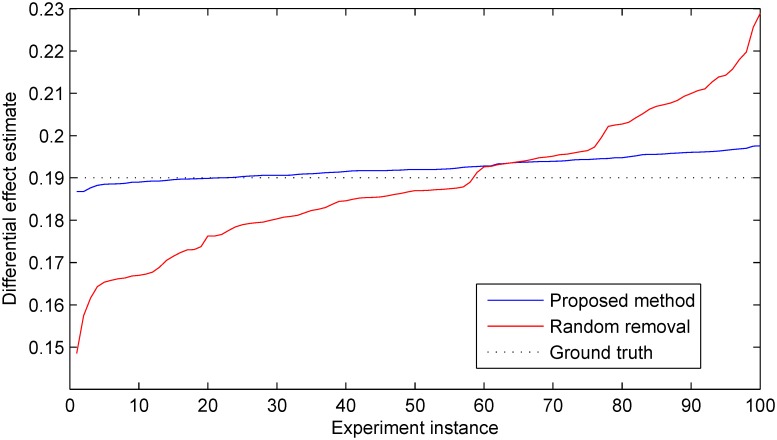
Estimates of the differential effect of treatment produced by the proposed method and the random selection-based baseline across 100 simulated trials. The estimates are shown ordered in magnitude for easier visualization. In all trial instances the reduction in the total number of participants of *n* = 70 (i.e. approximately 39% of the total cohort size of 180) was performed after *k* = 50 time steps. Following the removal time step, data accrual from the selected participants was discontinued but the data collected from them before that was still used in the final outcome analysis. Both the proposed method and the random selection-based baseline exhibited no bias, with a statistically insignificant error and the accuracy of within 1% from the ground truth. However the proposed method demonstrated far superior precision as readily observed from the plot; also see Figs 5–7.

As expected, averaged over 100 instances of the simulated experiment there was no significant difference in the accuracy of the estimate of differential effects in the treatment group relative to the control group between the two sample size reduction methods. In both cases the average accuracy was correct to within 1% with a statistically insignificant error from the ground truth. However an examination of the corresponding estimate precisions readily reveals a different picture. In particular across different experiment instances the proposed method results in approximately 70% lower standard deviation of the target estimate; this is illustrated in Fig 4. This finding illustrates with clarity the argument we put forward in the preceding sections. By performing sample selection in a manner which takes into account all available information, the statistically best decision can be made about which specific samples can be removed so as to ensure that in any particular case the associated information loss is minimized. In the experiments which follow, we sought to examine in detail how different parameters of a trial affect the precision improvement achieved with the proposed method.

### Experiment 2: Effects of timing

As in the previous experiment, we used the data generation model from *Experimental setup* and compared the proposed method with random sample selection in the context of a single sample size reduction step. Our aim in this experiment was to investigate the effect that the timing of participant removal, that is, the cessation of the corresponding data accrual, has on the precision gained by using the proposed method. Hence we performed simulations for three different time steps at which sample size reduction was performed, *k* = 30,50,70. All the other parameters were kept constant—the number of participants removed was set to *n* = 50 (i.e. approximately 28% of the total cohort size of 180), and the observability parameter which was set to *ϕ* = 0.01. As before the final outcomes were analysed after *k*_max_ = 100 time steps using the method described in [16], and for each combination of parameters 100 simulations were performed.

**Fig 5:**
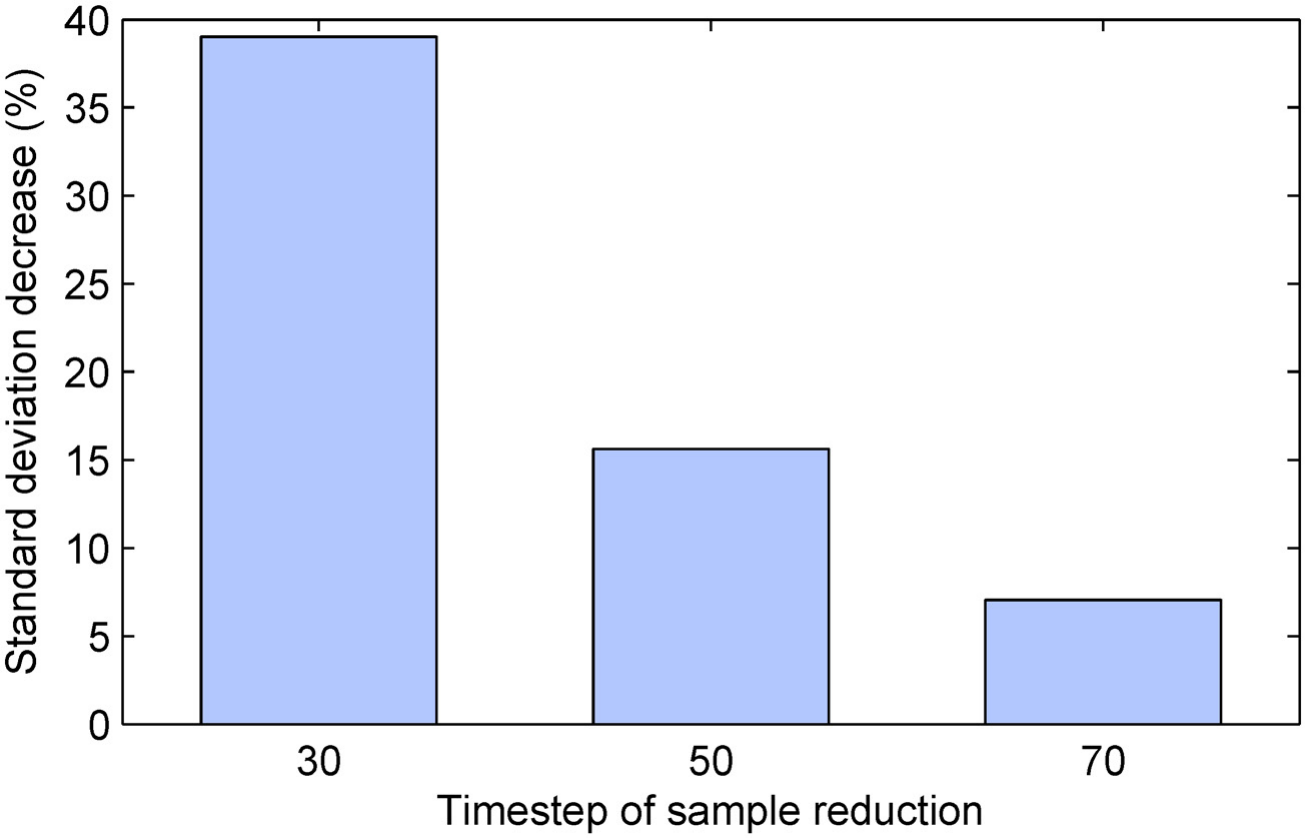
The impact of sample size reduction timing on the precision of the estimate of the differential effect of treatment. Plotted is the decrease in the standard deviation of the estimate across different simulated trial instances (in %, relative to the random selection-based baseline). In all cases the proposed method demonstrates a substantial improvement over the baseline. Greatest improvement is achieved for early sample size adjustment. Also see Fig 4.

Much like before and conforming to predictions from theory, we found no differential effect between the average estimates produced by the proposed method and the naïve random selection-based approach—in both cases the average accuracy was correct to within 1% with a statistically insignificant error from the ground truth. On the other hand the difference in precision was again stark and offered interesting insight into the proposed method. Our findings are summarized by the plot in Fig 5 which shows the reduction in the standard deviation of the estimate of the differential effect of treatment, across different simulation instances. Firstly, observe that in all cases, that is, regardless of the time step at which sample size reduction was performed, the proposed method achieved superior results over the naïve alternative. Secondly, it is immediately apparent that the greatest benefit was found when sample size reduction was performed earliest (i.e. after *k* = 30 time steps). In this case the standard deviation of the estimate was reduced extensively, by approximately 39%. Lower but still substantial reduction of approximately 16% was achieved when sample removal was performed half-way though the duration of the simulated trials. When the removal was delayed to after 70% of the duration of the trial, the standard deviation reduction was still significant at approximately 7%.

The observed behaviour of our algorithm is unsurprising and could be readily predicted from theory.

### Experiment 3: Effects of size adjustment

In this experiment our aim was to investigate the effect that the magnitude of sample size reduction (i.e. the number of participants for whom data accrual is discontinued) has on the precision gained by using the proposed method. The experiment was set up in the same manner as *Experiment 2* with the exception that now the time step at which sample size reduction was performed was set to *k* = 50 and kept at this value in all simulation instances, while the number of participants who were removed from the continued accrual of data was treated as an independent variable, *n* = 30, 50, 70. As before 100 simulations were performed with each combination of parameters.

**Fig 6:**
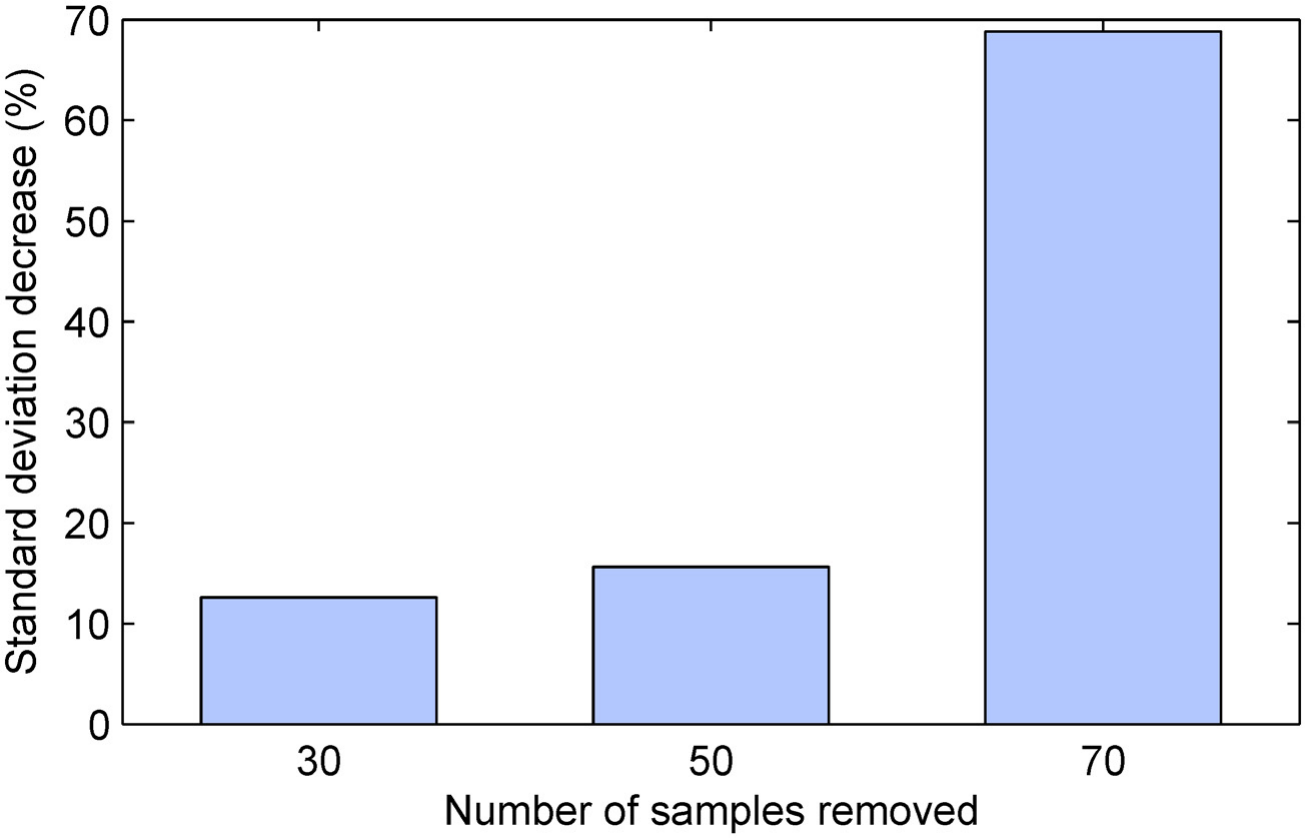
The impact of sample size reduction magnitude on the precision of the estimate of the differential effect of treatment. Plotted is the decrease in the standard deviation of the estimate across different simulated trial instances (in %, relative to the random selection-based baseline). In all cases the proposed method demonstrates a substantial improvement over the baseline. Greatest improvement is achieved for the removal of a large number of participants. Also see Fig 4.

As in the previous experiments, the average accuracy of the estimate of the differential effects of the treatment intervention over control was the same regardless of the sample selection strategy employed, but the corresponding precisions differed significantly. Our findings are summarized by the plot in Fig 6. Again, it is worth starting with the most obvious observation, which is that in all cases the proposed methodology resulted in an improvement. The greatest improvement was observed in the case when the sample size reduction was most drastic, with the proposed algorithm achieving nearly 70% lower standard deviation than the random selection-based alternative. This is of course consistent with our theoretical argument and supportive of the propositions put forward in the preceding sections—the more the sample size is reduced, the greater becomes the advantage of using all available information to make the adjustment targeted to the specific cohort.

### Experiment 4: Effects of perceptibility

In *Belief refinement* we highlighted the importance of *ϕ*, the observability parameter of the model used to generate trial data. Recall that this parameter was introduced to model the ease with which a participant can gauge the effects of the administered treatment i.e. the participant’s ability to observe an improvement or deterioration of the outcome of interest. The significance that this inherent nature of the studied phenomenon has in the context of the proposed sample size reduction methodology can be readily appreciated by observing that the proposed approach derives key power from the stratification of patients based on their responses to the auxiliary questionnaire (described in *Auxiliary data collection*). Consider the extreme case when perceptibility vanishes i.e. when *ϕ* = 0. In this case the collected auxiliary data is effectively random and as such contributes no useful information to guide the selection of participants which are to be removed from the trial. More generally, it can be said that the lower the perceptibility of the outcome, the less there is to be gained from using the proposed algorithm as compared to simple random selection.

**Fig 7:**
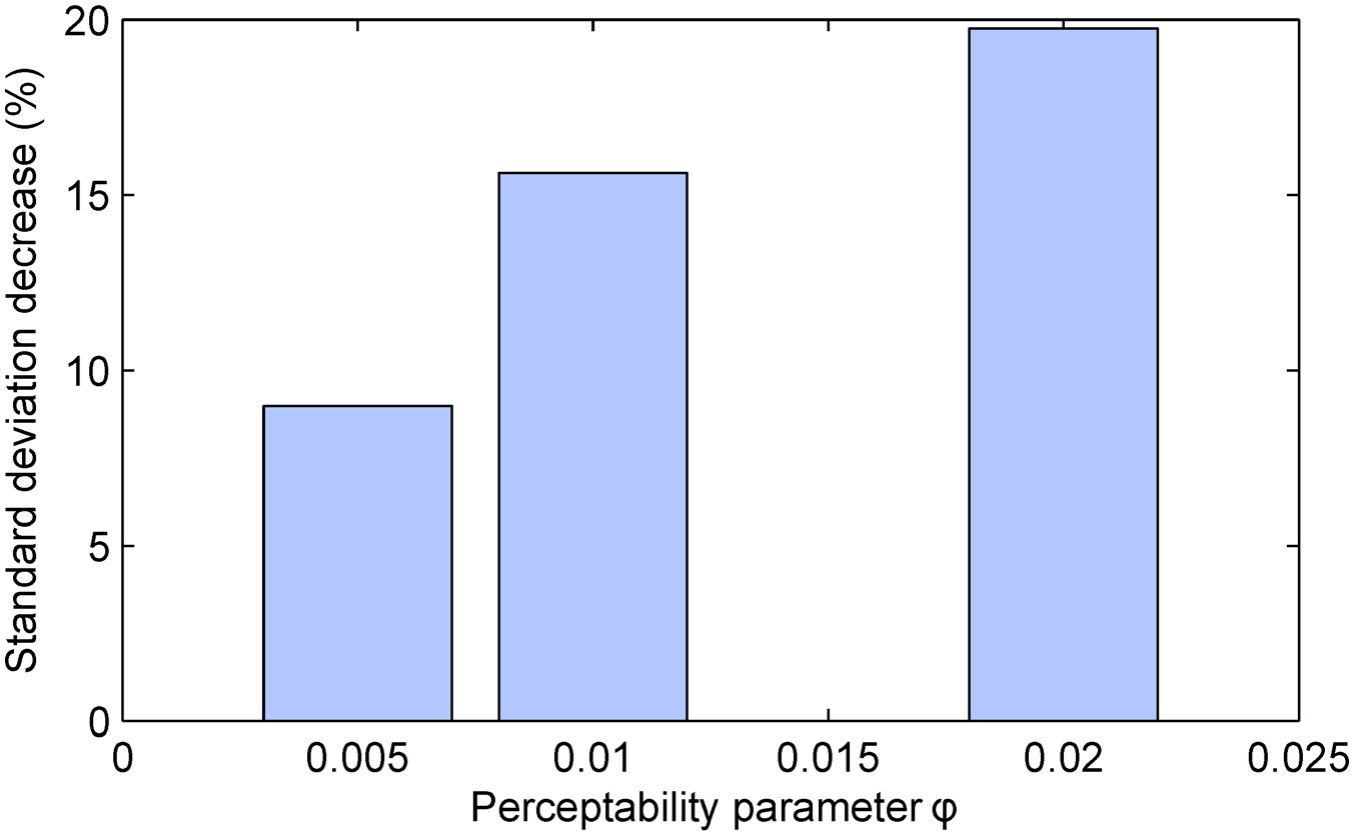
The impact of outcome perceptibility on the precision of the estimate of the differential effect of treatment. Plotted is the decrease in the standard deviation of the estimate across different simulated trial instances (in %, relative to the random selection-based baseline). In all cases the proposed method demonstrates a substantial improvement over the baseline. Greatest improvement is achieved for trials with high outcome perceptibility. Also see Fig 4.

Following the design adopted in the previous experiments, we performed 100 simulated trials for each combination of parameter values, while keeping all of the trial variables fixed except for the observability parameter. In addition to the previously used value of *ϕ* = 0.01, we examined the performance benefit of using our algorithm when observability was halved (*ϕ* = 0.005) and doubled (*ϕ* = 0.02). In all cases the number of removed participants was set to *n* = 50 which was performed after *k* = 50 time steps. The results are summarized in Fig 7 and can be seen to match our theoretical expectations.

### Experiment 5: Sequential adjustment

In the final experiment we report, we adopted a different experimental design than in the previous experiments. Specifically, in the previous experiments sample size reduction was understood to result in the cessation of data accrual for the removed trial participants, but the data accrued before their removal was still used in the final analysis of the trial outcomes. In contrast, in this experiment all data for the removed participants, including the data accrued before removal, is discarded for the final analysis. This design captures those trial instances when the studied treatment exhibits cumulative effects which complicate the analysis of outcomes observed at different points in time.

Following the same practice as in the previous experiments, we used the data generation model described in *Experimental setup*. However unlike before in this experiment sample size reduction was not performed at a single point in time. Rather a single participant was removed after each time step. Clearly this is not intended to replicate any realistic scenario which could be encountered in clinical practice. Though artificial in this sense, this experiment is useful as an analytical tool in the study of the behaviour of the proposed algorithm.

**Fig 8:**
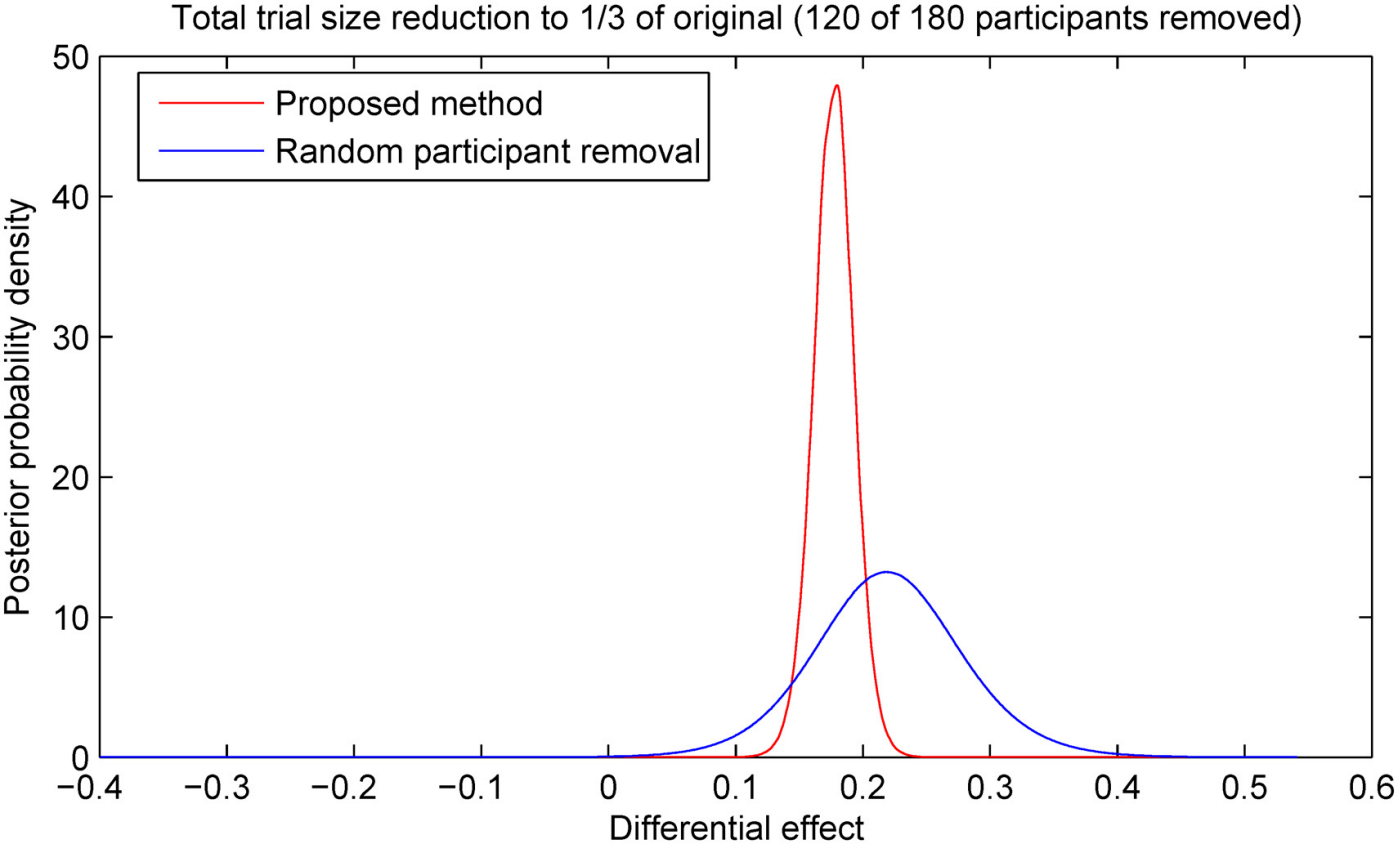
Posterior distributions of the differential effect of treatment after the removal of 120 participants i.e. after 120 time steps; see Eqs (32)–(42). The method introduced in this paper results in a much more certain estimate of the differential, as witnessed by the highly peaked distribution, than does random selection.

It is insightful to start with a look at the impact that the removal of a part of the cohort has on the estimate of the differential effect of the treatment intervention. A typical result is illustrated in Fig 8. This plot shows the posterior distributions of the differential effect of the treatment inferred after the removal of 120 individuals obtained using the proposed method (red line) and random selection (blue line). In both cases the posteriors were computed as described in [16]. The most notable difference between the two distributions is in their spreads i.e. the associated uncertainties. Specifically, the proposed method results in a much more peaked posterior, that is, a much more confident estimate. In comparison, the posterior obtained using random selection is much broader, admitting a lower degree of certainty associated with the corresponding estimate.

**Fig 9:**
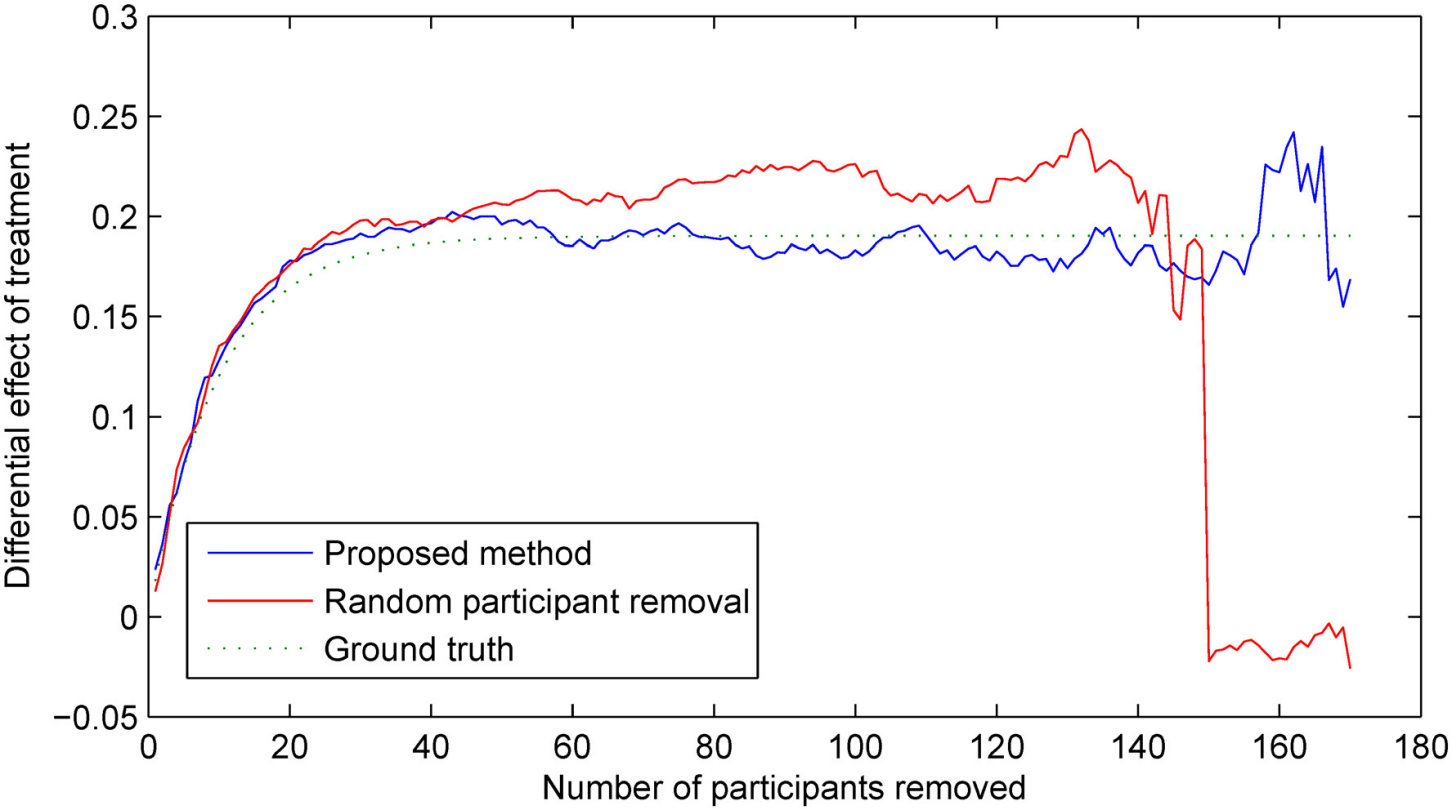
The *maximum a posteriori* estimates of the differential effect of treatment during the course of the trial as an increasing number of participants is removed. The blue line shows the estimates obtained using the proposed method, while the red line shows the estimates obtained using random selection. Also shown is the ‘ground truth’ (green dotted line), that is, the actual differential effect computed exactly from the setup of the experiment (see *Experimental setup*).

The accuracy of the two methods is better assessed by observing their behaviour over time. The plot in Fig 9 shows the *maximum a posteriori* estimates of the differential effect of treatment obtained using the two methods during the course of the trial. Also shown is the ‘ground truth’, that is, the actual differential effect which we can compute exactly from the setup of the experiment (see *Experimental setup*). In the early stages of the trial, while the magnitude of the accumulated effect is small and the number of participants large, the two estimates are virtually indistinguishable, and they follow the ground truth plot closely. As expected, as the number of participants removed increases both estimates start to exhibit greater stochastic perturbations. However, both the accuracy (that is, the closeness to the ground truth) and the precision (that is, the magnitude of stochastic variability) of the proposed method can be seen to show superior performance—its *maximum a posteriori* estimate follows the ground truth more closely and fluctuates less than the estimate obtained when random selection is employed instead. It is also important to observe the rapid degradation of performance of the random selection method as the number of remaining participants becomes small, which is not seen in the proposed method. This too can be expected from the theoretical argument put forward in *Sub-group selection*—the statistically optimal choice of the sub-group from which participants are removed ensures that the posterior is not highly dependent on a small number of samples which would make it highly sensitive to the change in sample size.

**Fig 10:**
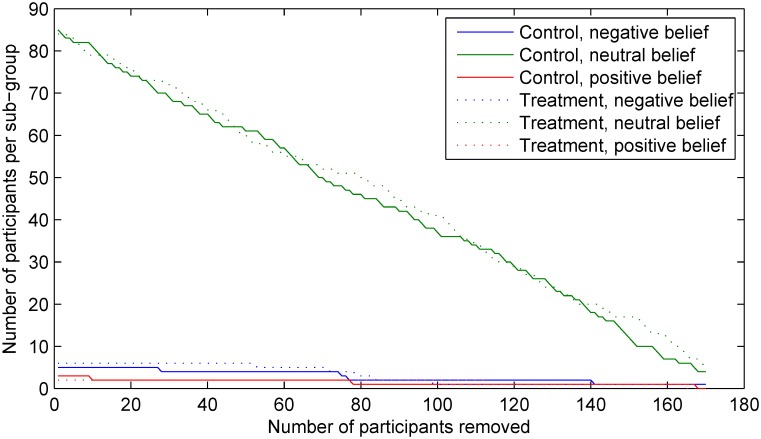
The changes in the sample sizes within each of the six participant sub-groups (using the stratification described in *Matching sub-groups outcome model* and adopted from [16]) observed in our experiment using random selection based participant removal. Note the predictable outcome of the method, which results in a linear decrease of sample size for all sub-groups, and the contrasting behaviour of our approach in Fig 11.

**Fig 11:**
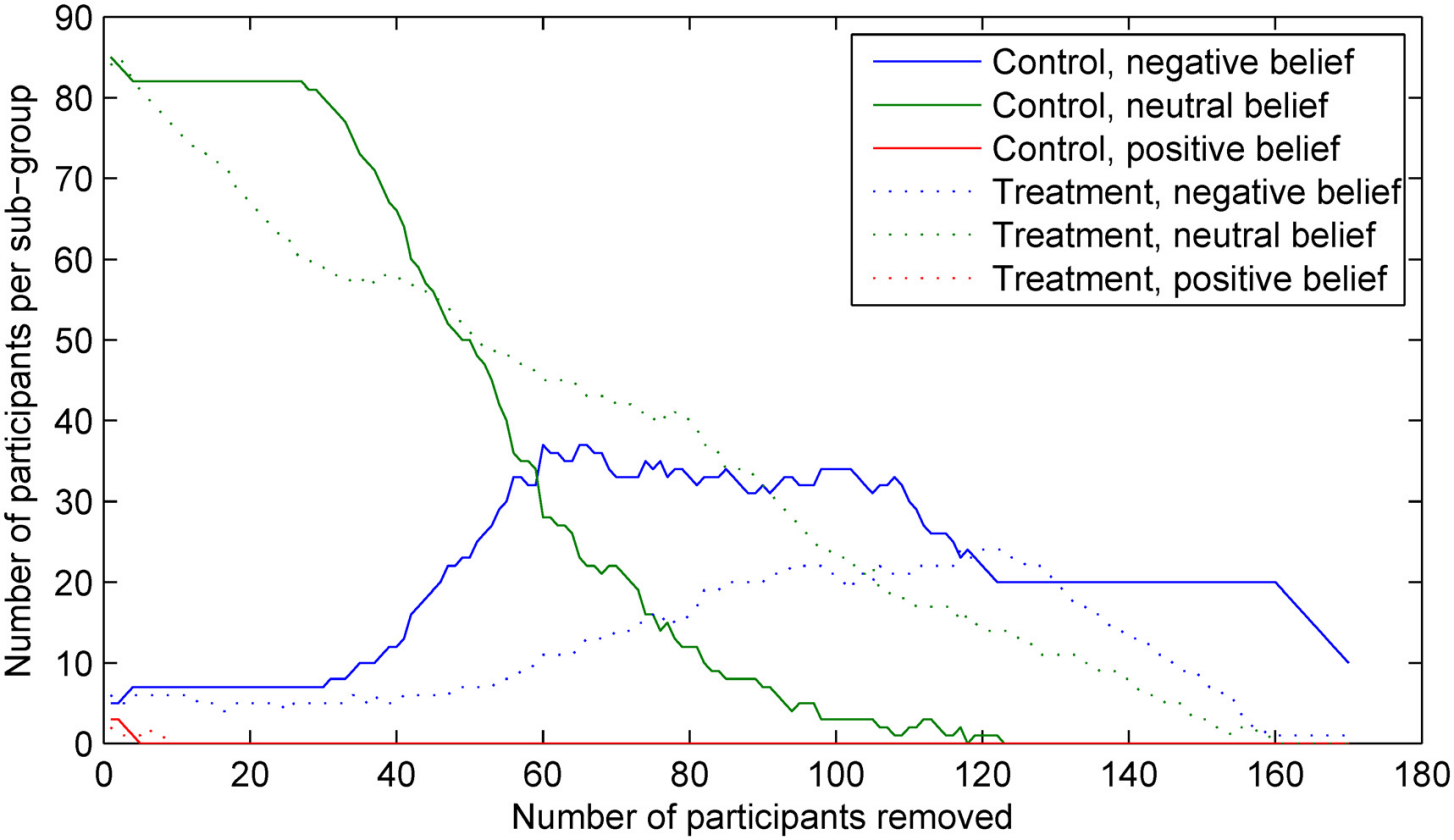
The changes in the sample sizes within each of the six participant sub-groups (using the stratification described in *Matching sub-groups outcome model* and adopted from [16]) observed in our experiment using the proposed method. Compare with Fig 10 and note the data-specific behaviour of our approach.

Lastly, it is interesting to observe the differences between the changes in the sample sizes within each sub-group using the two approaches. This is illustrated using the plots in Figs 10 and 11. As expected, when random participant removal is employed, the sizes of all sub-groups decrease roughly linearly (save for stochastic variability), as shown in Fig 10. In contrast, the sub-group size changes effected by the proposed method show more complex structure, governed by the specific values of the belief and effect variables in our experiment, as shown in Fig 11. It is particularly interesting to note that the size changes are not only non-linear, but also non-monotonic. For example, the relative size of the control sub-group which includes individuals which correctly identified their group assignment (i.e. the sub-group *G*_*C*−_) begins to increase notably after the removal of 30 participants and starts to decrease only after the removal of further 78 participants.

#### Summary and conclusions

In this paper we introduced a novel method for clinical trial adaptation. Our focus was on adaptation by amending sample size. In contrast to all previous work in this area, the problem we considered was not *when* sample size should be adjusted but rather *which* particular individuals should be removed from the trial once the decision of sample reduction is made. Thus, our method is not an alternative to the current state-of-the-art, but rather a complementary element of the same framework. Our approach is based on the adopted stratification recently proposed for the analysis of trial outcomes in the presence of imperfect blinding. This stratification is based on the trial participants’ responses to a generic auxiliary questionnaire that allows each participant to express belief concerning his/her intervention assignment (treatment or control). Extensive experiments on a simulated trial were used to illustrate the effectiveness of our method.
